# Modeling *PTEN* overexpression-induced microcephaly in human brain organoids

**DOI:** 10.1186/s13041-021-00841-3

**Published:** 2021-08-30

**Authors:** Navroop Dhaliwal, Wendy W.Y. Choi, Julien Muffat, Yun Li

**Affiliations:** 1 Program in Developmental and Stem Cell Biology, The Hospital for Sick Children, 686 Bay Street, Toronto, M5G 0A4 ON Canada; 2 Department of Molecular Genetics, University of Toronto, 1 King’s College Circle, Toronto, M5S 1A8 ON Canada; 3 Program in Genetics and Genome Biology, The Hospital for Sick Children, 686 Bay Street, Toronto, M5G 0A4 ON Canada; 4 Program in Neurosciences and Mental Health, The Hospital for Sick Children, 686 Bay Street, Toronto, M5G 0A4 ON Canada

**Keywords:** PTEN, AKT, Brain organoids, Human pluripotent stem cells, Neural precursors, Microcephaly, Neurodevelopmental disorder

## Abstract

The phosphatase and tensin homolog (PTEN) protein, encoded by the *PTEN* gene on chromosome 10, is a negative regulator of the phosphoinositide 3-kinase (PI3K) signaling pathway. Loss of *PTEN* has been linked to an array of human diseases, including neurodevelopmental disorders such as macrocephaly and autism. However, it remains unknown whether increased dosage of *PTEN* can lead to human disease. A recent human genetics study identifies chromosome 10 microduplication encompassing *PTEN* in patients with microcephaly. Here we generated a human brain organoid model of increased *PTEN* dosage. We showed that mild *PTEN* overexpression led to reduced neural precursor proliferation, premature neuronal differentiation, and the formation of significantly smaller brain organoids. *PTEN* overexpression resulted in decreased AKT activation, and treatment of wild-type organoids with an AKT inhibitor recapitulated the reduced brain organoid growth phenotypes. Together, our findings provide functional evidence that *PTEN* is a dosage-sensitive gene that regulates human neurodevelopment, and that increased *PTEN* dosage in brain organoids results in microcephaly-like phenotypes.

**Supplementary Information:**

The online version contains supplementary material available at 10.1186/s13041-021-00841-3.

Loss of function mutations in the *PTEN* tumor suppressor gene are implicated in a wide spectrum of human diseases. In the central nervous system, loss of *PTEN* leads to brain cancers, as well as non-malignant conditions including macrocephaly, autism, and epilepsy [1]. However, the impact of *PTEN* overexpression on human health remains largely unknown. Partial trisomy of chromosome 10, including distal 10q where *PTEN* resides, has been found in patients with severe developmental disorders including craniofacial malformations. Recently, a 10q23.31 microduplication has been identified in patients with autosomal dominant primary microcephaly [2]. *PTEN* is one of the 3 genes duplicated in this region, and is hypothesized to be the causal gene for the condition. However, the functional impact of *PTEN* overexpression on human neurodevelopment has not been experimentally examined.

The advent of human pluripotent stem cells (hPSCs) and 3-dimensional brain organoid technologies provides a new avenue to investigate human neurodevelopment in vitro. We and others have previously utilized these tools to model primary microcephaly caused by genetic mutations [3–6] and environmental factors such as the Zika virus [7–9]. To understand the role of *PTEN* loss of function in regulating human neurodevelopment, we have previously generated *PTEN* knockout hPSCs [10]. *PTEN* knockout brain organoids are significantly larger in size, mimicking the macrocephalic conditions seen in patients with *PTEN* loss of function mutations.

Here, we generated an hPSCs-derived brain organoid model of mild *PTEN* overexpression (PTEN-OE) to study the effect of increased *PTEN* dosage on neurodevelopment. Wild-type WIBR3 hPSCs were transduced with lentivirus expressing a PTEN-GFP fusion cDNA [10]. GFP-positive subclones were selected (Fig. 1A) and examined for their *PTEN* expression levels using quantitative RT-PCR. We identified three subclones with mild overexpression of *PTEN* (Fig. 1B) and further verified their PTEN protein levels using immuno-blotting (Fig. 1C, D). Three subclones of the same parental WIBR3 hPSCs were used as controls. We next generated forebrain organoids by directed differentiation [4, 10]. Equal numbers of control and PTEN-OE hPSCs were aggregated to form embryoid bodies of similar size and morphology (Fig. 1F and Additional file 1: Figure S1A, B). These embryoid bodies were cultured in neural differentiation medium and embedded in Matrigel droplets to form forebrain organoids. We observed that the growth of PTEN-OE organoids was slower compared to controls, and PTEN-OE organoids were significantly smaller at 5–6 weeks (Fig. 1E, F). *PTEN* overexpression in embryoid bodies and organoids was confirmed using quantitative RT-PCR (Additional file 1: Figure S1C). To investigate whether the reduced organoid growth was linked to altered cellular proliferation, we next performed immuno-staining for Ki67. Within the ventricular zone where neural precursors reside, fewer Ki67-positive cells were observed in 3-week-old PTEN-OE organoids compared to their isogenic controls (Fig. 1G, H). This reduction coincided with decreased neural precursor markers (SOX2, TBR2) as measured by quantitative RT-PCR in 3-week-old organoids (Fig. 1I). Because reduced neural precursor proliferation may lead to cell cycle exit and differentiation, we next examined the level of neuronal markers DCX and CTIP2 (Fig. 1J–L). At 3 weeks, PTEN-OE organoids expressed increased levels of DCX and CTIP2 transcripts (Fig. 1J). Immuno-staining further confirmed that while few DCX- and CTIP2-positive neurons were present in control organoids, they were more abundant in PTEN-OE organoids (Fig. 1K, L). Therefore, our data indicates that increased dosage of *PTEN* reduces neural precursor proliferation, promotes premature neuronal differentiation, and results in the formation of significantly smaller brain organoids, mimicking the microcephalic condition seen in patients with 10q23.31 microduplication.

**Fig. 1:**
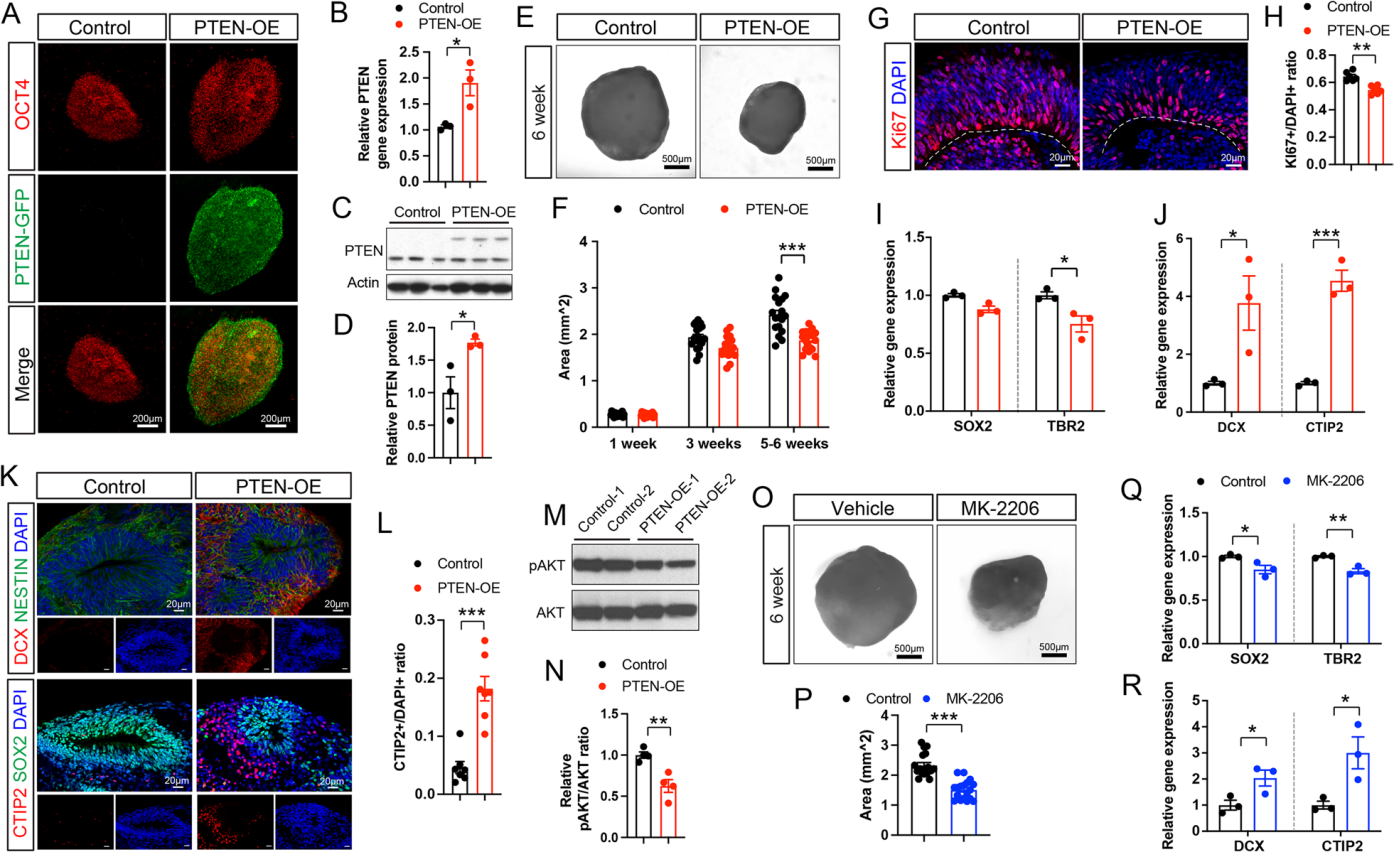
PTEN-OE brain organoids model human microcephaly. **A** Immuno-staining of control and PTEN-OE hPSCs for markers of pluripotency (OCT4). Presence of GFP indicates overexpression of PTEN-GFP fusion protein. **B** Quantitative RT-PCR shows increased expression of *PTEN* in PTEN-OE hPSCs. Each data point represents one independent hPSC line (n = 3 for each group). **C** Immuno-blotting analysis shows increased total PTEN protein level (higher molecular weight band indicates PTEN-GFP fusion protein) in PTEN-OE hPSCs. **D** Quantification of total PTEN protein levels (endogenous PTEN and PTEN-GFP) in control and PTEN-OE hPSCs, normalized to Actin. Each data point represents one independent hPSC line (n = 3 for each group). **E** Representative images of control and PTEN-OE organoids at 6 weeks. **F **Quantification of control and PTEN-OE organoid size at around 1 week (day 7-10, 3 weeks (day 21-24), and 5-6 weeks (day 35-42). Each data point represents the area of a single organoid. N = 18 for each group at each time point from 3 hPSC lines and 2 independent differentiation experiments. ANOVA revealed significant effects of age (F_2,102_=669.4, *p* < 0.0001), genotype (F_1,102_=34.65, *p* < 0.0001), and the interaction between the two (F_2,102_=12.20, *p* < 0.0001). **G** and **H** Representative images (**G)** and quantification (**H)** of KI67 immuno-staining in 3-week-old control and PTEN-OE brain organoids. White dashed lines indicate the apical edge of the ventricular zone. Each data point represents one hPSC line in an independent differentiation, from the average of measurement from 3 organoids. N = 6 for each group from 3 hPSC lines and 2 independent differentiation experiments. **I** and **J** Quantitative RT-PCR analysis of neural precursor markers SOX2, TBR2 (**I)** and neuronal markers DCX, CTIP2 (**J)** in 3-week-old control and PTEN-OE brain organoids. Each data point represents one independent hPSC line (n = 3 for each group). **K** Representative images of immuno-staining for DCX and NESTIN (upper panels), CTIP2 and SOX2 (lower panels) in 3-week-old control and PTEN-OE organoids. **L** Quantification of CTIP2 immuno-staining in 3-week-old control and PTEN-OE brain organoids. Each data point represents one hPSC line in an independent differentiation, from the average of measurement from 3 organoids. N = 6 for each group from 3 hPSC lines and 2 independent differentiation experiments. **M** Immuno-blotting analysis shows reduced phospho-AKT protein level in 3-week-old PTEN-OE brain organoids generated from hPSC line Control-1, Control-2, PTEN-OE-1, and PTEN-OE-2. **N** Quantification of immuno-blotting results (Figure  1M and Additional file 1: Figure S1D) shows reduced phospho-AKT to total AKT ratio in 3-week-old PTEN-OE brain organoids. Each data point represents one hPSC line in an independent differentiation. N = 4 for each group from 3 hPSC lines and 2 independent experiments. **O** Representative images of 6-week-old brain organoids treated with vehicle or AKT inhibitor MK-2206 (100 nM). **P** Size quantification of vehicle and MK-2206 treated brain organoids at 5-6 weeks. Each data point represents the area of a single organoid. N = 18 for each group from 3 hPSC lines and 2 independent differentiation experiments. **Q** and **R** Quantitative RT-PCR analysis of neural precursor markers SOX2, TBR2 (**Q**) and neuronal markers DCX, CTIP2 (**R)** in vehicle and MK-2206 treated 6-week-old brain organoids. Each data point represents one independent hPSC line (n = 3 for each group).Results are mean ± SEM. *p<0.05, **p<0.01, ***p<0.001

To investigate the molecular signaling that contributed to the cellular phenotypes, we next performed immuno-blotting for activated (phosphorylated) AKT. Consistent with its function as a negative regulator of the PI3K-AKT pathway, we found that PTEN-OE organoids had reduced level of phospho-AKT (Fig. 1M, N and Additional file 1: Figure S1D). Because PTEN has functions dependent and independent of AKT activation, we investigated whether AKT inhibition in control organoids could recapitulate the PTEN-OE phenotype. We generated brain organoids from control hPSCs in the continuous presence of vehicle or 100 nM MK-2206, a known AKT inhibitor, starting from day 1 of embryoid body formation. We have previously used MK-2206 to inhibit the aberrant AKT activation in *PTEN* knockout brain organoids and showed that it was effective in restoring normal organoid growth [10]. Here we found that MK-2206 treated organoids were significantly smaller than vehicle treated controls at 5–6 weeks (Fig. 1O, P). Quantitative RT-PCR demonstrated that MK-2206 treatment led to decreased level of neural precursor markers (SOX2, TBR2) and increased presence of neuronal markers (DCX, CTIP2) (Fig. 1Q, R). The chronic treatment of MK-2206 mimicked the systemic overexpression of *PTEN* in patients and the in vitro organoid cultures. We further investigated the impact of short-term treatment of MK-2206 on hPSCs-derived neural precursors. We found that neural precursors cultured in the presence of growth factors (FGF2 and insulin) and 100 nM MK-2206 for 7 days showed reduced AKT activity, as measured by the level of phospho-AKT (Additional file 1: Figure S2A, B) and phospho-S6, a downstream target of the AKT-mTOR pathway (Additional file 1: Figure S2A and C). While this treatment did not reduce SOX2 transcript level, it led to a significant decrease in TBR2, suggesting TBR2-positive intermediate progenitors may be more acutely vulnerable to AKT inhibition (Additional file 1: Figure S2D). Together, these findings suggest that increased dosage of *PTEN* leads to microcephaly in vitro by reducing AKT pathway activity.

In summary, our study provides functional evidence that increased dosage of *PTEN* contributes to impaired neurodevelopment in vitro. Given the known role of loss of *PTEN* in disorders including autism and macrocephaly, this new insight places *PTEN* amongst other dosage-sensitive causal genes (such as *MECP2*, *SHANK3*, *SCN2A*, *UBE3A*) for neurodevelopmental disorders. These findings are consistent with human genetics findings that aberrant activation and inhibition of the PI3K-AKT signaling pathway are both implicated in abnormal brain formation [11–14]. Future studies utilizing the hPSCs-derived brain organoid platform may provide additional insights into the disease etiology and therapeutic options for *PTEN*-related neurodevelopmental disorders.

The WIBR3 human embryonic stem cell line was approved for use by the Stem Cell Oversight Committee of the Canadian Institutes of Health Research, and the Research Ethics Board of the Hospital for Sick Children.

## Supplementary Information

**Additional file 1: Figure S1. **Analysis of PTEN-OE embryoid bodies and brain organoids. **Figure S2. **Short-term MK-2206 treatment of human neural precursors. **Table S1. **Antibody information. **Table S2. **Primer information.

## Data Availability

All data generated or analyzed during this study are included in this article.

## Abbreviations

PTEN: Phosphatase and tensin homolog
AKT: Ak strain transforming, also known as protein kinase B
PI3K: Phosphoinositide 3-kinase
hPSCs: Human pluripotent stem cells
OE: Overexpression
RT-PCR: Reverse transcription polymerase chain reaction
