# Supplementary material for: Modeling PTEN overexpression-induced microcephaly in human brain organoids
Source: Mol Brain. 2021 Aug 30;14:131. doi: 10.1186/s13041-021-00841-3 (PMC8404342; doi:10.1186/s13041-021-00841-3)
Supplement: Supplementary file 1 — Additional file 1: Figure S1. Analysis of PTEN-OE embryoid bodies and brain organoids. Figure S2. Short-term MK-2206 treatment of human neural precursors. Table S1. Antibody information. Table S2. Primer information. [file 13041_2021_841_MOESM1_ESM.pdf]

Additional Information for

Modeling PTEN overexpression induced microcephaly in human brain organoids

Navroop Dhaliwal[1,5], Wendy W.Y. Choi[1,2,3,5], Julien Muffat[1,2,4], and Yun Li[1,2]*

Affiliations:

1. Program in Developmental and Stem Cell Biology, The Hospital for Sick Children, 686 Bay Street, Toronto, ON M5G 0A4, Canada.

2. University of Toronto, Department of Molecular Genetics, 1 King's College Circle, Toronto, ON M5S 1A8, Canada.

3. Program in Genetics and Genome Biology, The Hospital for Sick Children, 686 Bay Street, Toronto, ON M5G 0A4, Canada.

4. Program in Neurosciences and Mental Health, The Hospital for Sick Children, 686 Bay Street, Toronto, ON M5G 0A4, Canada.

5. These authors contributed equally.

*Correspondence to: yun.li@sickkids.ca (Y.L.).

This file includes:

Additional materials and methods

Figure S1, S2, Table S1, S2

References for Additional information

Additional Materials and Methods

Human pluripotent stem cell culture

Human embryonic stem cell line WIBR3 was previously described (1) and cultured on mitomycin-C (Sigma, M4287) inactivated mouse embryonic fibroblasts (MEFs) in medium containing DMEM/F12 (Thermo, 11330032), 15% fetal bovine serum (Hyclone, SH30084), 5% knockout serum replacement (KSR, Thermo, 10828028), 1% non-essential amino acids (Sigma, M7145), 2mM Glutamax (Thermo, 35050061), 0.1mM beta-mercaptoethanol (Sigma, M6250), and 4ng/ml bFGF (Thermo, PHG0263). Cultures were passaged manually or with 1mg/ml collagenase type IV (Thermo, 17104019) every 5-7 days. This and other cell lines were routinely tested for mycoplasma negativity.

Brain organoid culture

Brain organoids were generated from WIBR3 human embryonic stem cells as previously described (1), with modifications. Briefly, human embryonic stem cells were dissociated from MEFs using collagenase type IV (Thermo, 17104019), and further separated from residual MEFs by gravity separation, before trypsinization to generate single cells. A total of 9000 cells were then plated into each well of a PrimeSurface v-bottom 96-well plate (S-Bio, MS-9096VZ) to form single embryoid bodies, in medium containing DMEM/F12 (Thermo, 11330032), 20% KSR (Thermo, 10828028), 2mM Glutamax (Thermo, 35050061), 1% non-essential amino acids (Sigma, M7145), 50nM beta-mercaptoethanol (Sigma, M6250), 4ng/ml bFGF (Thermo, PHG0263), and 2.5uM dorsomorphin (Stemgent, 04-0024). Rho-associated protein kinase (ROCK) inhibitor Y27632 (50uM,

Stemgent, 04-0012) was included in the first 24 hours. Embryoid bodies were maintained in 96-well plates for 6 days, then transferred to ultra-low-attachment 24-well plates (Corning, 3473), in “neural glial medium” (NGD 0.5X) as described previously (2). On days 10-12, embryoid bodies were embedded in droplets of Matrigel (Corning, 356234), and were allowed to gel at 37C. Droplets were cultured in stationary condition in ultra-low-attachment 6-well plates (Corning, 3471) for 4 days, followed by transfer to an orbital shaker rotating continuously at 150rpm. For AKT inhibitor experiments, embryoid bodies were treated with vehicle or 100nM MK-2206 (Selleckchem, S1078) continuously, from day 1 to 6 weeks.

Neural precursor culture

Differentiation of human embryonic stem cells to neural precursors in 2D adherent culture was performed as previously described (2, 3). Briefly, hPSCs were passaged onto Matrigel-coated dishes using PBS without Ca^{2+}/Mg^{2+}, filtered through a 40um cell strainer (Falcon, 352340) to remove MEFs, and cultured directly in NGD 0.5X plus 2.5uM dorsomorphin (Stemgent, 04-0024), 10ng/mL bFGF (Thermo, PHG0263) and human insulin (additional 20ug/mL, Sigma, 9278) until super-confluent. bFGF and additional insulin were removed after a week, and NGD 0.5X plus dorsomorphin was replaced every day for 10 days. Cells were subsequently passaged with Accutase (Thermo, A1110501) when rosette lawns were observed throughout the culture. ROCK inhibitor Y27632 (Stemgent, 04-0012, 10uM) was added to the medium during the first 3 passages. After the first passage, neural precursors were expanded and maintained in NGD 0.5X plus 10ng/ml bFGF and 20ug/mL human insulin. For AKT inhibitor experiments, passage 9 to

11 neural precursors were treated with vehicle or 100nM MK-2206 (Selleckchem, S1078) for 7 days.

Lentivirus production and transduction

FUW-GFP-PTEN lentivirus construct was previously reported (1, 4). VSVG-coated lentiviruses were generated in HEK293 cells. Briefly, HEK293 cells were transfected using X-tremeGENE 9 (Sigma, 6365809001), with a mixture of lentiviral construct and packaging plasmids. Culture medium was changed 12 hours after and collected 96 hours after transfection. Virus-containing medium was filtered through 0.45um filter (Fisher, 14-386-77) and concentrated via ultracentrifugation. For transduction experiments, hPSCs were first dissociated from MEFs using collagenase type IV (Thermo, 17104019), and further dissociated using Trypsin/EDTA (Thermo, 25300054) to generate single cells. hPSCs were plated onto Matrigel-coated dishes and fed mTeSR medium (Stem Cell Technologies, 85875) plus 10uM ROCK inhibitor Y27632 (Stemgent, 04-0012), and lentiviral particles. hPSCs were subsequently passaged as single cells at clonal density to MEFs, and single clones were picked and expanded. Expression of lentiviral-transgene was evaluated using GFP fluorescence, quantitative RT-PCR and immuno-blotting.

Histology and imaging

Cells and tissues were fixed with 4% (w/v) paraformaldehyde (Sigma, 158127) in PBS. Paraffin sections were prepared from organoids. Following membrane permeabilization with PBS containing 0.3% Triton x-100 (Sigma, T8787), cells and organoid sections were blocked with 3% normal donkey serum (Sigma, S30-M). Primary antibodies were against

SOX2, TBR2, Ki67, DCX, CTIP2, and visualized by secondary antibodies conjugated with Alexa 488, 594, followed by counter-staining with DAPI (Thermo, D3571). Detailed primary and secondary antibody information is described in Table S1. Fluorescent images of immuno-staining were captured on a Zeiss LSM-700 confocal microscope. Quantifications of KI67, CTIP2 immuno-staining, and normalization to DAPI staining were performed using ImageJ.

RNA extraction, reverse transcription and quantitative PCR

Cells and organoids were homogenized and total RNA extracted using the RNeasy kit (Qiagen, 74106) following manufacturer's instructions. Total RNA concentrations were measured using NanoDrop ND-1000 or DeNovix DS-11+ spectrophotometer. RNA was reverse transcribed into cDNA using Superscript IV reverse transcriptase (Thermo, 18090200) with random hexamer primers, or a mix of oligo $d(T)_{20}$ primers and random hexamer primers, according to manufacturer's instructions. Transcript representation was determined by quantitative PCR using PowerUp SYBR Green PCR mix (Applied Biosystems, A25778), with primer pairs against PTEN, SOX2, TBR2, DCX, CTIP2 and GAPDH. Cellular RNA raw Ct values were normalized to GAPDH. Detailed primer information is described in Table S2. For the purpose of quantitative analysis, transcripts not detected were assigned a Ct value of 40.

Protein purification and immuno-blotting

Total protein was extracted from cells and tissues using RIPA lysis buffer containing 50mM Tris-HCL pH 7.4, 150mM NaCl, 0.25% deoxycholic acid 1% NP-40, 1mM EDTA

(Millipore, 20-188), with the addition of protease inhibitor cocktail (Roche, 11836170001), phosphotase inhibitor cocktail 2 and 3 (Sigma, P5726, P0044). Total protein from the supernatant was measured using BCA protein assay (Pierce, 23209). Primary antibodies were against Actin, PTEN, phospho-AKT, AKT, phospho-S6, S6, and were visualized with HRP-conjugated secondary antibodies, using a LumiGlo Chemiluminescent Kit (KPL, KP-54-71-02), or a Radiance Plus Chemiluminescent kit (Azure Biosystems, AC2103), in accordance with the manufacturer's instructions. Detailed primary and secondary antibody information is described in Table S1. Membranes blotted for phospho-proteins were stripped and re-probed with antibodies against total proteins. Membranes blotted for PTEN were stripped and re-probed for Actin. Values for phospho-proteins were normalized to total proteins, and PTEN was normalized to Actin.

Statistics

All data values were presented as mean +/- SEM. Student's t tests were applied to data with two groups. ANOVA analyses were used for comparisons of data with greater than two groups. Post hoc group comparisons were performed with Turkey test. A value of $p<0.05$ was considered significant.

Figure S1

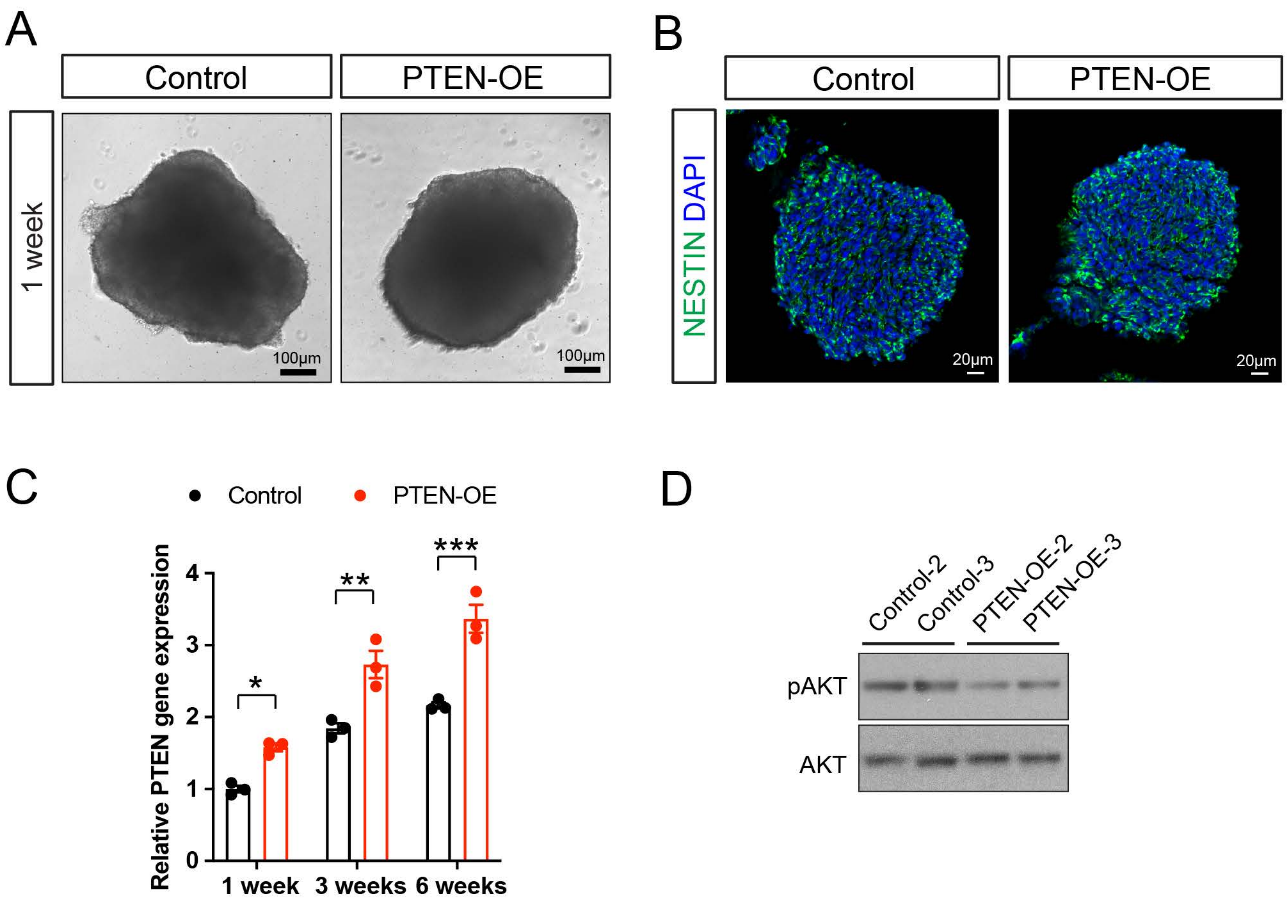
A
Control
PTEN-OE
1 week
100μm
100μm
B
Control
PTEN-OE
NESTIN DAPI
20μm
20μm
C
Control
PTEN-OE
Relative PTEN gene expression
0
1
2
3
4
*
**
***
1 week
3 weeks
6 weeks
D
Control-2
Control-3
PTEN-OE-2
PTEN-OE-3
pAKT
AKT

Additional Figure Legends

Figure S1. Analysis of PTEN-OE embryoid bodies and brain organoids.

(A) Representative images of embryoid bodies generated from control and PTEN-OE hPSCs at 1 week, showing their similar size and morphology at this stage.

(B) Immuno-staining for NESTIN in control and PTEN-OE embryoid bodies at 10 days, showing similar level of neural induction.

(C) Quantitative RT-PCR shows increased expression of *PTEN* in PTEN-OE organoids at 1, 3, and 6 weeks. Each data point represents one independent hPSC line. n=3 for each group at each time point. ANOVA revealed significant effects of age ($F_{2,12}$=79.20, p<0.0001) and genotype ($F_{1,12}$=82.97, p<0.0001).

(D) Immuno-blotting analysis shows reduced phospho-AKT protein level in 3-week-old PTEN-OE brain organoids generated from hPSC line Control-2, Control-3, PTEN-OE-2, and PTEN-OE-3.

Results are mean +/- SEM. *p<0.05, **p<0.01, ***p<0.001.

Figure S2

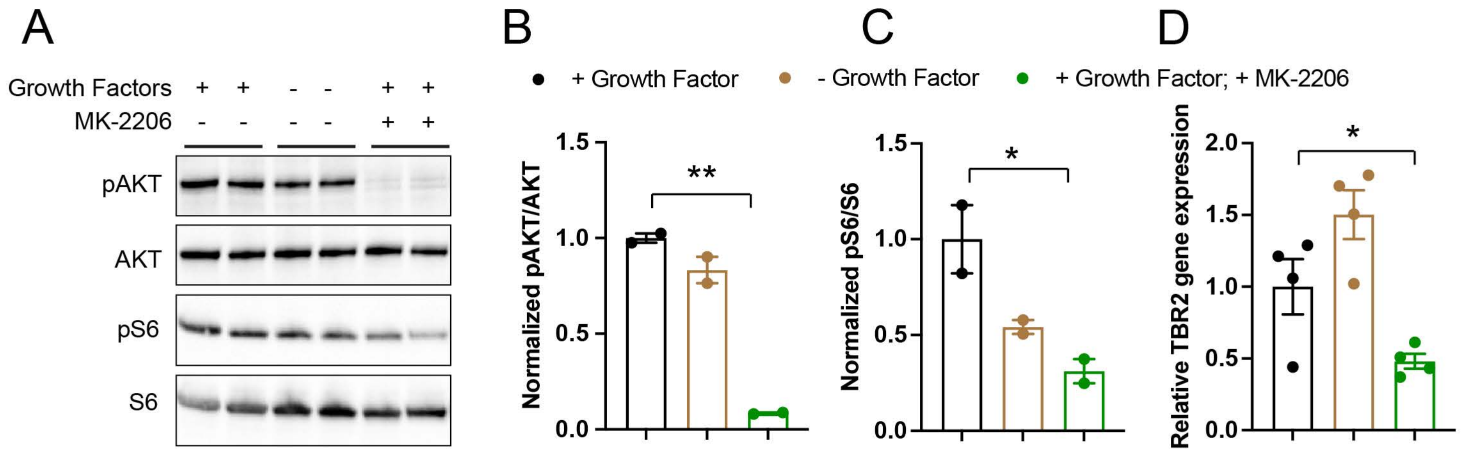
A
B
C
D
Growth Factors + + - - + +
MK-2206 - - - - + +
pAKT
AKT
pS6
S6
+ Growth Factor
- Growth Factor
+ Growth Factor; + MK-2206
Normalized pAKT/AKT
Normalized pS6/S6
Relative TBR2 gene expression
**
*
*
0.0
0.5
1.0
1.5
2.0

Figure S2. Short-term MK-2206 treatment of human neural precursors.

(A-C) Immuno-blotting analysis for phospho-AKT (A-B) and phospho-S6 (A and C) shows reduced activation of AKT and S6 in control hPSCs-derived neural precursors treated with the AKT inhibitor MK-2206 (100nM for 7 days). Each data point represents one independent hPSC line. N=2 for each group. ANOVA revealed significant effects of treatment for phospho-AKT ($F_{2,3}$=133.2, p=0.0012), and phospho-S6 ($F_{2,3}$=9.950, p=0.0474).

(D) Quantitative RT-PCR analysis shows reduced TBR2 transcript level in control neural precursors treated with MK-2206. Each data point represents one hPSC line in an independent differentiation. N=4 for each group from 2 hPSC lines and 2 independent differentiation experiments. ANOVA revealed significant effects of treatment ($F_{2,9}$=13.05, p=0.0022).

Results are mean +/- SEM. *p<0.05, **p<0.01.

Table S1. Antibody information.

S1a. Antibodies for immuno-staining

Antibody	Vendor	Catalog #	Species	Dilution
CTIP2	Abcam	ab18465	Rat	1:500
DCX (Doublecortin)	Santa Cruz	sc-8066	Goat	1:500
KI67	Dako	M7240	Mouse	1:100
NESTIN	Millipore	MAB5326	Mouse	1:300
OCT4	Abcam	GT486	Mouse	1:100
SOX2	R&D Systems	AF2018	Goat	1:300
Goat IgG Alexa 594	Thermo	A11058	Donkey	1:500
Goat IgG Alexa 488	Thermo	A11055	Donkey	1:500
Rat IgG Alexa 594	Thermo	A21209	Donkey	1:500
Mouse IgG Alexa 594	Thermo	A21203	Donkey	1:500
Mouse IgG Alexa 488	Thermo	A21202	Donkey	1:500

S1b. Antibodies for immuno-blotting

Antibody	Vendor	Catalog #	Species	Dilution
Phospho-AKT	Cell Signaling	4058	Rabbit	1:1000
AKT	Cell Signaling	9272	Rabbit	1:1000
Phospho-S6	Cell Signaling	4858	Rabbit	1:1000
S6	Cell Signaling	2217	Rabbit	1:1000
PTEN	Cell Signaling	9559	Rabbit	1:1000
Actin	Sigma	A2228	Mouse	1:1000
Rabbit IgG HRP	Thermo	31460	Goat	1:1000
Mouse IgG HRP	Thermo	31430	Goat	1:1000

Table S2. Primer information.

**Gene**	**Primer (forward)**	**Primer (reverse)**
CTIP2	GAGTACTGCGGCAAGGTGTT	TAGTTGCACAGCTCGCACTT
DCX	TCCCGGATGAATGGGTTGC	GCGTACACAATCCCCTTGAAGTA
GAPDH	CGTGGAAGGACTCATGACCA	CAGTCTTCTGGGTGGCAGTGA
PTEN	TGGATTCGACTTAGACTTGACCT	TGGCGGTGTCATAATGTCTTTC
SOX2	GCCGAGTGGAAACTTTTGTCG	GGCAGCGTGTACTTATCCTTCT
TBR2	CACCGCCACCAAACTGAGAT	CGAACACATTGTAGTGGGCAG

Reference

1. Li Y, *et al.* (2017) Induction of Expansion and Folding in Human Cerebral Organoids. *Cell stem cell* 20(3):385-396 e383.
2. Li Y, *et al.* (2019) Genome-wide CRISPR screen for Zika virus resistance in human neural cells. *Proc Natl Acad Sci U S A*.
3. Li Y, *et al.* (2013) Global transcriptional and translational repression in human-embryonic-stem-cell-derived Rett syndrome neurons. *Cell stem cell* 13(4):446-458.
4. Fricano CJ, *et al.* (2014) Fatty acids increase neuronal hypertrophy of Pten knockdown neurons. *Front Mol Neurosci* 7:30.
